# Efficacy of more intensive lipid-lowering therapy on cardiovascular diseases: a systematic review and meta-analysis

**DOI:** 10.1186/s12872-020-01567-1

**Published:** 2020-07-13

**Authors:** Hsin-Yin Hsu, Chien-Ju Lin, Yu-Shan Lee, Ting-Hui Wu, Kuo-Liong Chien

**Affiliations:** 1 Department of Family Medicine, MacKay Memorial Hospital, No. 92, Section 2, Zhongshan North Road, Taipei City, 10449 Taiwan; 2 Institute of Epidemiology and Preventive Medicine, College of Public Health, National Taiwan University, No.17, Xu-Zhou Rd, Taipei City, 10055 Taiwan; 3 Department of Family Medicine, Hsinchu MacKay Memorial Hospital, No. 690, Section 2, Guangfu Road, East District, Hsinchu City, 30071 Taiwan; 4 Department of Internal Medicine, National Taiwan University Hospital, No. 7, Zhongshan S. Rd., Zhongzheng Dist, Taipei City, 10055 Taiwan

**Keywords:** Intensive lipid-lowering, Primary prevention, Cardiovascular outcome, All-cause mortality

## Abstract

**Background:**

Cardiovascular disease is the leading cause of morbidity and mortality with incidence rates of 5–10 per 1000 person-years, according to primary prevention studies. To control hyperlipidemia—a major risk factor of cardiovascular disease—initiation of lipid-lowering therapy with therapeutic lifestyle modification or lipid-lowering agent is recommended. Few systematic reviews and meta-analyses are available on lipid-lowering therapy for the primary prevention of cardiovascular diseases. In addition, the operational definitions of intensive lipid-lowering therapies are heterogeneous. The aim of our study was to investigate whether intensive lipid-lowering therapies reduce greater cardiovascular disease risks in primary prevention settings.

**Methods:**

MEDLINE, EMBASE, and Cochrane Library databases were searched from inception to March 2019 for randomized controlled trials. We used random effects model for overall pooled risk ratio (RR) estimation with cardiovascular events of interest and all-cause mortality rate for the intensive lipid-lowering group using the standard lipid-lowering group as the reference. The Cochrane Risk of Bias Tool was used for quality assessment.

**Results:**

A total of 18 randomized controlled trials were included. The risk reductions in cardiovascular outcomes and all-cause mortality associated with more intensive vs. standard lipid-lowering therapy across all trials were 24 and 10%, respectively (RR 0.76, 95% confidence interval 0.68–0.85; RR 0.90, 95% confidence interval 0.83–0.97); however, the risk reduction varied by baseline LDL-C level in the trial. A greater risk reduction was noted with higher LDL-C level. Intensive lipid-lowering for coronary heart disease protection was more pronounced in the non-diabetic populations than in the diabetic populations.

**Conclusions:**

More intensive LDL-C lowering was associated with a greater reduction in risk of total and cardiovascular mortality in trials of patients with higher baseline LDL-C levels than less intensive LDL-C lowering. Intensive lipid-lowering was associated with a significant risk reduction of coronary heart disease and must be considered even in the non-diabetic populations.

## Background

Cardiovascular diseases (CVD), particularly atherosclerotic CVD, are responsible for almost half of all non-communicable disease deaths over the past decade. Coronary heart diseases (CHD), account for the largest proportion of CVD-related mortality. Although CHD can be attributed to numerous risk factors, hyperlipidemia plays the most critical role [1]. To control hyperlipidemia, especially elevated low-density lipoprotein cholesterol (LDL-C), initiation of lipid-lowering therapy with therapeutic lifestyle modification or lipid-lowering agent is recommended [1]. Guidelines have suggested distinct targets of atherogenic lipoprotein level and varying intensities of lipid-lowering agent use according to stratified risks for coronary heart diseases using cardiovascular disease risk calculators [2–5]. In primary prevention settings, therapeutic lifestyle modification is the principal strategy to improve lipid profiles for populations with low-to-medium risk of atherosclerotic cardiovascular diseases. For high cardiovascular risk populations, i.e., those with diabetes or several high-risk conditions, a more intensive lipid-lowering strategy is usually recommended, including a combination of lipid-lowering agents with therapeutic lifestyle changes to reach a stricter target of atherogenic lipoprotein levels [3–5]. However, the extent of LDL-C treatment and indication of intensive treatment have been under discussion in primary prevention settings [6–8]. Meanwhile, current epidemiological evidence demonstrates inconclusive effect of intensive lipid treatment on all-cause mortality prevention. The heterogeneity of operational definitions of intensive lipid-lowering therapies is also noted [4, 9, 10]. The limited evidence elucidates the effect modifications of sex, age, and diabetes status between intensive lipid treatment and coronary heart disease prevention [11]. Thus, the first aim of our study was to investigate whether intensive lipid-lowering therapies reduce greater CHD or all-cause mortality risks in primary prevention settings. The second aim was to determine whether the different definitions of intensive lipid treatment and different baseline LDL-C levels are the source of heterogeneity of CHD protective effect from intensive lipid treatment. The final aim was to evaluate whether factors, including sex, age, and diabetes status, are important effect modifiers between intensive lipid treatment and coronary heart disease prevention.

## Methods

### Data sources and study selection

Our systemic review and meta-analysis was conducted to evaluate the efficacy of intensive lipid-lowering treatment on CVD. Results were reported following the Preferred Reporting Items for Systematic reviews and Meta-Analyses (PRISMA) statement [12]. MEDLINE, EMBASE, and Cochrane Library databases were searched from inception to March 2019 for randomized controlled trials (RCTs). Our search strategy was discussed and revised with a librarian. The key words used were as follows: cholesterol, low-density lipoprotein cholesterol, lipid-lowering, coronary artery diseases, heart attacks, myocardial infarctions, cardiovascular disease, and randomized controlled trial. The complete search strategy used in MEDLINE is shown in Table S2. After the initial search, three authors (C.-J.L., Y.-S.L., and T.-H.W.) independently performed the initial title and abstract screening to exclude irrelevant articles. Conflicts were resolved through discussion with the fourth author (H.-Y.H.). Two independent reviewers (H.-Y.H. and C.-J.L.) completed the detailed reading and assessed the eligibility of each article.

The inclusion criteria were as follows (1) RCTs, (2) reported hard cardiovascular outcomes, (3) population without clinically evident coronary artery disease, and (4) one treatment group received more intensive lipid-lowering therapy with a control group. The exclusion criteria were as follows: (1) 3- or 4-arm studies and (2) head-to-head comparisons of different single pharmacologic interventions in the same drug category.

### Study outcomes and data extraction

The outcomes of interest were cardiovascular events including fatal or nonfatal myocardial infarction, unstable angina requiring hospitalization, or coronary revascularization. All-cause mortality rate was also the result of interest. We obtained the absolute event numbers in each lipid-lowering group.

Heterogeneity was noted in the definitions of intensive lipid-lowering versus standard therapy. We then depicted intensive lipid-lowering by (1) more potent pharmacologic agent, (2) more LDL-C reduction percentage, (3) lower absolute LDL-C level, and (4) stricter lipid treatment guideline.

For each included trial, three authors (C.-J.L., Y.-S.L., and T.-H.W.) independently performed the data extraction, including first author’s name, publication year, description of the study population (sample size), trial duration, event numbers in the intensive/standard lipid-lowering groups, effect size whether with hazard ratios or risk ratios (RR) with 95% confidence intervals (CI), mean age of the population, proportion of women and diabetes mellitus participants, baseline LDL-C level, and degree of LDL-C reduction (both in absolute value and percentage) in each group. All data abstraction was verified by another author (H.-Y.H.).

### Risk of bias and quality assessment

Quality assessment was performed using the Cochrane Risk of Bias Tool revised version (RoB 2.0) to evaluate potential risk of bias [13]. This approach specifies three levels of quality: “high,” “some concerns,” and “low” across five domains. We reviewed the included studies and assessed the randomization process, deviations from the intended interventions, missing outcome data, measurement of the outcome, and selection of the reported result. Disagreements were resolved through discussions with other authors.

### Statistical analysis

A random effects model was used for overall pooled RR estimation with cardiovascular events of interest and all-cause mortality rate for the intensive lipid-lowering group by using the standard lipid-lowering group as the reference. We used Cochrane Q test to evaluate the heterogeneity between studies and *I*^2^ for the magnitude of heterogeneity (*I*^2^ values): 25, 50, and 75% represent mild, moderate, and high heterogeneity, respectively. Subgroup analyses were conducted using different definitions of intensive versus standard lipid-lowering mentioned above and different stratifications of baseline LDL-C level (< 130 mg/dL, between 130 and 160 mg/dL, and > 160 mg/dL) [14]. Random-effect meta-regressions with different covariates, such as baseline LDL-C level, age, proportion of women, and diabetes mellitus, were carried out to explore the potential effect modifier in the association between the different lipid-lowering strategies and outcomes of interest [15]. We performed sensitivity analyses to judge the robustness of the summary RR we estimated. A potential publication bias and probable small study effect were examined by visual inspection of funnel plot and Egger’s linear regression test. Results are presented as RR with 95% CIs. Statistical significance was set at *p* < 0.05. R 3.5.1(R Core Team, 2018) was used for all statistical analyses. We resolved the discrepancy in literature search, trial and data extraction, and quality assessment after discussion and consensus.

## Results

**Fig. 1:**
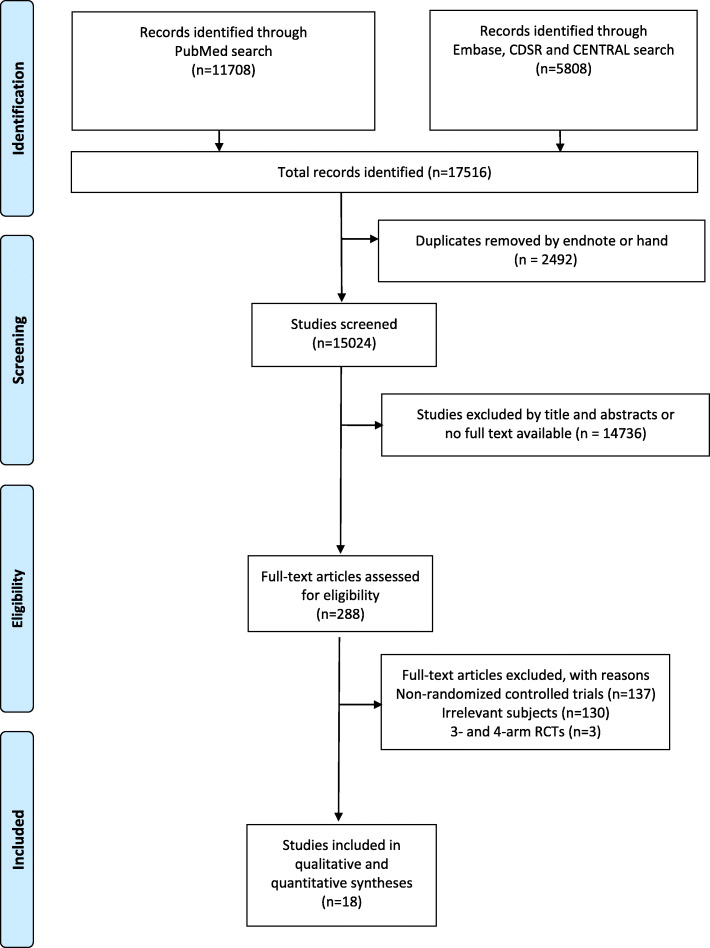
PRISMA study selection flow diagram. CDSR, Cochrane Database of Systematic Reviews; CENTRAL, Cochrane Central Register of Controlled Trials

PRISMA flow chart is shown in Fig. 1. A total of 15,024 studies were initially found, of which 288 were completely screened after deleting the duplicates and excluding irrelevant studies. Eighteen RCTs were included and appraised after ruling out (1) the interventions or outcomes not of interest and (2) non-RCTs. Among the included studies, 18 trials were with coronary event analysis (*N* = 103,864); 15 trials, total mortality event analysis (*N* = 93,215); 16 trials, lipid-lowering drug (11 statin trials [16–26], 1 cholestyramine resin trial [18, 27], 1 fibrate trial [28], 1 gemfibrozil trial [29], 1 combination therapy trial [30]) vs. placebo; two trials, lipid-lowering drug vs. usual care (statin trials [31, 32]; usual care was defined as medical care with nutrition education and lipid-lowering medication prescribed by health care providers); and 1 trial, absolute LDL-C level (a statin trial [33]). The characteristics of each trial are provided in Table 1. A total of 103,864 participants were randomly allocated to the more intensive lipid-lowering (*N* = 52,008) and control (*N* = 51,856) groups. Mean follow-up duration was 4.0 years (range 1–7.4 years). Mean baseline LDL-C level was 144.7 mg/dL (range 106.1–205.3 mg/dL). Greater LDL-C reduction (19.0–49.1%) was noted in the intensive lipid-lowering group than in the standard lipid-lowering group (− 6.5–15.3%) at 1–2 years of follow-up. Mean age of participants was 60 years (range 30–80 years). The proportion of women varied from 0 to 87.4% within studies. Six trials had total diabetes participants, and the prevalence of diabetes population for the rest of the trials was between 1.2 and 24.6%.

**Table 1:** Characteristics of randomized controlled trials investigating efficacy of intensive lipid lowering in primary prevention settings

Study	Country	Participants	Intervention	Compariason	Baseline LDL-C level, mean (SD)^**a**^	Main findings [n (%), intervention vs. comparison]
LRC-CPPT, 1984 [27]	North America	3806 participants, mean follow-up duration 7.4 years, age 30–59 years, women proportion 0%	Bile acid sequestrant cholestyramine resin 24 g/day (*n* = 1906)	Placebo (*n* = 1900)	205.3 mg/dL	**LDL-C reduction percentage**^b^22.4% vs. 2.8% **CHD event***n* = 155 (8.1%) vs. *n* = 187 (9.8%) **All-cause mortality***n* = 68 (3.6%) vs. *n* = 71 (3.7%)
HHS, 1987 [29]	Finland	4081 participants, mean follow-up duration 5.0 years, mean age 47.2 years, diabetes proportion 2.6%	Gemfibrozil 600 mg twice/day (*n* = 2051)	Placebo (*n* = 2030)	189.2 mg/dL	**LDL-C reduction percentage** 8.7% vs. -2.9% **CHD event***n* = 56 (2.7%) vs. *n* = 84 (4.1%)
ACAPS, 1994 [16]	United States	919 participants, mean follow-up duration 3.0 years, mean age 61.7 years, women proportion 48.5%, diabetes proportion 2.3%	Lovastatin 20–40 mg/day (*n* = 460)	Placebo (*n* = 459)	155.6 mg/dL	**LDL-C reduction percentage** 28.0% vs. -3.5% **CHD event***n* = 5 (1.1%) vs. *n* = 5 (1.1%)**All-cause mortality***n* = 1 (0.2%) vs. *n* = 8 (1.7%)
WOSCOPS, 1995 [17]	Scotland	6595 participants, mean follow-up duration 4.9 years, mean age 55.2 years, women proportion 0%, diabetes proportion 1.2%	Pravastatin 40 mg/day (*n* = 3302)	Placebo (*n* = 3293)	192.0 mg/dL	**LDL-C reduction percentage** 26.0% vs. 1.6% **CHD event***n* = 173 (5.2%) vs. *n* = 248 (7.5%) **All-cause mortality***n* = 106 (3.2%) vs. *n* = 135 (4.1%)
AFCAPS/TexCAPS, 1998 [18]	United States	6605 participants, mean follow-up duration 6.5 years, mean age 58.0 years, women proportion 15.1%, diabetes proportion 2.3%	Lovastatin 20–40 mg/day (*n* = 3304)	Placebo (*n* = 3301)	150.0 mg/dL	**LDL-C reduction percentage** 25.0% vs. -1.5% **CHD event***n* = 116 (3.5%) vs. *n* = 183 (5.5%)
Sasaki et al., 2002 [31]	Japan	1085 participants, mean follow-up duration 6.5 years, mean age 58.0 years, women proportion 15.1%, diabetes proportion 2.3%	Pravastatin 10–20 mg/day (*n* = 587)	Usual care ^c^ (*n* = 498)	200.0 mg/dL	**LDL-C reduction percentage** 22.8% vs. 14.0% **CHD event***n* = 5 (0.9%) vs. *n* = 13 (2.6%) **All-cause mortality***n* = 4 (0.7%) vs. *n* = 2 (0.4%)
ASCOT-LLA, 2003 [17]	United Kingdom, Ireland	10,305 participants, mean follow-up duration 3.3 years, mean age 63.0 years, women proportion 18.8%, diabetes proportion 24.6%	Atorvastatin 10 mg/day (*n* = 5168)	Placebo (*n* = 5137)	131.5 mg/dL	**LDL-C reduction percentage** 35.0% vs. 0.0% **CHD event***n* = 273 (5.3%) vs. *n* = 346 (6.7%) **All-cause mortality***n* = 185 (3.6%) vs. *n* = 212 (4.1%)
CARDS, 2004 [21]	United Kingdom, Ireland	2838 participants, mean follow-up duration 3.9 years, mean age 61.6 years, women proportion 32.0%, diabetes proportion 100%	Atorvastatin 10 mg/day (*n* = 1428)	Placebo (*n* = 1410)	117.6 mg/dL	**LDL-C reduction percentage** 38.8% vs. -2.6% **CHD event***n* = 62 (4.3%) vs. *n* = 92 (6.5%) **All-cause mortality***n* = 61 (4.3%) vs. *n* = 82 (5.8%)
Beishuizen et al., 2004 [20]	Netherlands	182 participants, mean follow-up duration 2.0 years, mean age 58.5 years, women proportion 52.8%, diabetes proportion 100%	Cerivastatin 0.4 mg/day (2001 replaced with Simvastatin 20 mg/day) (*n* = 103)	Placebo (*n* = 79)	137.3 mg/dL	**LDL-C reduction percentage** 25.0% vs. -6.5% **CHD event***n* = 0 (0.0%) vs. *n* = 4 (5.1%) **All-cause mortality***n* = 3 (2.9%) vs. *n* = 4 (5.1%)
FIELD, 2005 [28]	Australia, New Zealand and Finland	9795 participants, mean follow-up duration 5.0 years, mean age 62.2 years, women proportion 77.7%, diabetes proportion 100%	Fenofibrate 200 mg/day(*n* = 4895)	Placebo (*n* = 4900)	118.7 mg/dL	**LDL-C reduction percentage** 20.8% vs. 15.3% **CHD event***n* = 356 (7.3%) vs. *n* = 323 (6.6%) **All-cause mortality***n* = 454 (9.3%) vs. *n* = 508 (10.4%)
ASPEN, 2006 [22]	Multi-countries	1905 participants, mean follow-up duration 4.0 years, mean age 60.5 years, women proportion 52.8%, diabetes proportion 100%	Atorvastatin, 10 mg/day (*n* = 959)	Placebo (*n* = 946)	114.0 mg/dL	**LDL-C reduction percentage** 30.5% vs. 0.5% **CHD event***n* = 73 (7.6%) vs. *n* = 73 (7.7%) **All-cause mortality***n* = 44 (4.6%) vs. *n* = 41 (4.3%)
MEGA, 2006 [32]	Japan	7832 participants, mean follow-up duration 5.3 years, mean age 58.3 years, women proportion 67.5%, diabetes proportion 20.5%	Pravastatin 10–20 mg/day (*n* = 3866)	Usual care (*n* = 3966)	156.6 mg/dL	**LDL-C reduction percentage** 19.0% vs. 2.0% **CHD event***n* = 66 (1.7%) vs. *n* = 101 (2.5%) **All-cause mortality***n* = 55 (1.4%) vs. *n* = 79 (2.0%)
JUPITER, 2008 [23]	Multi-countries	17,802 participants, mean follow-up duration 1.9 years, mean age 66.0 years, women proportion 38.2%	Rosuvastatin 20 mg/day (*n* = 8901)	Placebo (*n* = 8901)	108.0 mg/dL	**LDL-C reduction percentage** 49.1% vs. -1.9% **CHD event***n* = 109 (1.2%) vs. *n* = 187 (2.1%) **All-cause mortality***n* = 198 (2.2%) vs. *n* = 247 (2.8%)
Heljic et al., 2009 [24]	Sarajevo	95 participants, mean follow-up duration 1.0 years, mean age 60.1 years, women proportion 57.9%, diabetes proportion 100%	Simvastatin 40 mg (*n* = 45)	Placebo (*n* = 50)	167.8 mg/dL	**LDL-C reduction percentage** 20.0% vs. 5.0%**CHD event***n* = 3 (6.7%) vs. *n* = 7 (14.0%)
SHARP, 2011 [30]	Multi-countries	9270 participants, mean follow-up duration 4.9 years, mean age 62.0 years, women proportion 37.4%, diabetes proportion 12.3%	Simvastatin 20 mg plus ezetimibe 10 mg/day (*n* = 4650)	Placebo (*n* = 4620)	107.1 mg/dL	**LDL-C reduction percentage** 39.0% vs. 0.7% **CHD event***n* = 213 (4.6%) vs. *n* = 230 (5.0%) **All-cause mortality***n* = 1142 (24.6%) vs. *n* = 1115 (24.1%)
HOPE-3, 2016 [25]	Multi-countries	12,705 participants, mean follow-up duration 5.6 years, mean age 65.8 years, women proportion 46.2%, diabetes proportion 5.8%	Rosuvastatin 10 mg/day (*n* = 6361)	Placebo (*n* = 6344)	127.8 mg/dL	**LDL-C reduction percentage** 31.0% vs. 1.5% **CHD event***n* = 105 (1.7%) vs. *n* = 140 (2.2%) **All-cause mortality***N* = 334 (5.3%) vs. *n* = 357 (5.6%)
EMPATHY, 2018 [33]	Japan	5042 participants, mean follow-up duration 3.1 years, mean age 63.1 years, women proportion 52.3%, diabetes proportion 100%	Target of LDL-C level lower than 70 mg/dL (*n* = 2518)	Target of LDL-C level lower than 100 mg/dL (*n* = 2524)	106.1 mg/dL	**LDL-C reduction percentage** 22.2% vs. 0.0% **CHD event***n* = 129 (5.1%) vs. *n* = 153 (6.1%) **All-cause mortality***N* = 41 (1.6%) vs. *n* = 34 (1.3%)
Kitas et al., 2019 [26]	United Kingdom	3002 participants, mean follow-up duration 2.5 years, mean age 61.0 years, women proportion 87.4%, diabetes proportion 100%	Atorvastatin 40 mg/day (*n* = 1504)	Placebo (*n* = 1498)	123.7 mg/dL	**LDL-C reduction percentage** 30.9% vs. 6.9% **CHD event***n* = 15 (1.0%) vs. *n* = 24 (1.6%) **All-cause mortality***N* = 25 (1.7%) vs. *n* = 27 (1.8%)

Unit conversion in LDL-C: 1 mmol/L = 38.6 mg/dL

*Abbreviations*: *LDL-C* Low-density lipoprotein cholesterol, *CHD* Coronary heart disease.

^a^The baseline LDL-C level is presented as the mean or median in the intervention group in each trial

^b^LDL-C reduction percentage is calculated by the difference of the LDL-C level between baseline LDL-C level and 1–2 year on-treatment or study closed LDL-C level

^c^Usual care indicates that the participants were under lipid lowering treatment with nutrition education and lipid lowering medication prescribed by health care providers’ professional decisions

### Cardiovascular outcomes

**Fig. 2:**
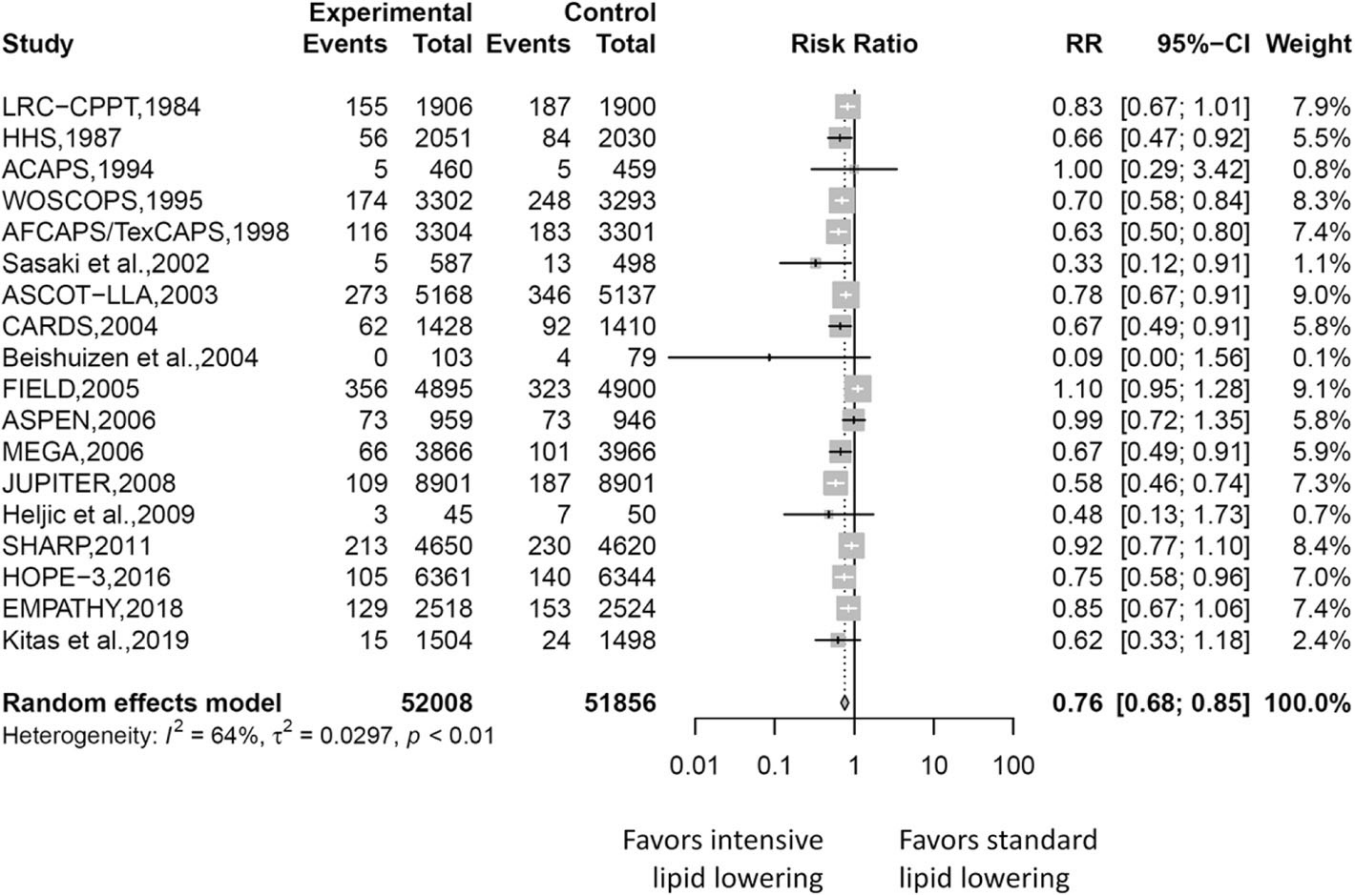
Forest plot of pooled RR of coronary events

Random-effect meta-analyses for pooled RRs for cardiovascular outcomes are demonstrated in Fig. 2. A total of 4315 cardiovascular events, including fatal/non-fatal myocardial infarction, unstable angina, and coronary revascularization, were identified. Of these cardiovascular outcomes, 1915 were found in the intensive lipid-lowering group and 2400 in the standard lipid-lowering group. An estimated 24% risk reduction in cardiovascular outcomes between the two lipid-lowering groups was found after pooling all study results (RR 0.76, 95% CI 0.68–0.85, *I*^2^ = 64%). Subgroup analyses of risk for coronary events of participants in the intensive lipid-lowering group by different definitions showed consistent results (Figure S1). The summary estimate revealed cardiovascular risk reduction of 23% in the pharmacologic agent vs. placebo group (RR 0.77, 95% CI 0.68–0.87, *I*^2^ = 67%), 44% in the pharmacologic agent vs. usual care group (RR 0.56, 95% CI 0.30–1.03, *I*^2^ = 43%), and 15% in the absolute LDL-C level group (RR 0.76, 95% CI 0.68–0.85, *I*^2^ = 64%), favoring more intensive lipid-lowering.

**Fig. 3:**
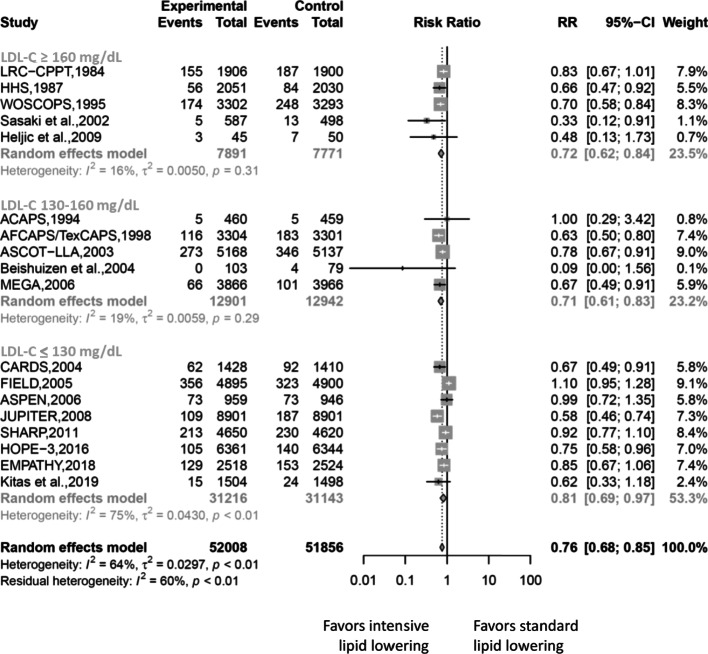
Forest plot of subgroup analyses of coronary event risks according to baseline LDL-C levels

**Fig. 4:**
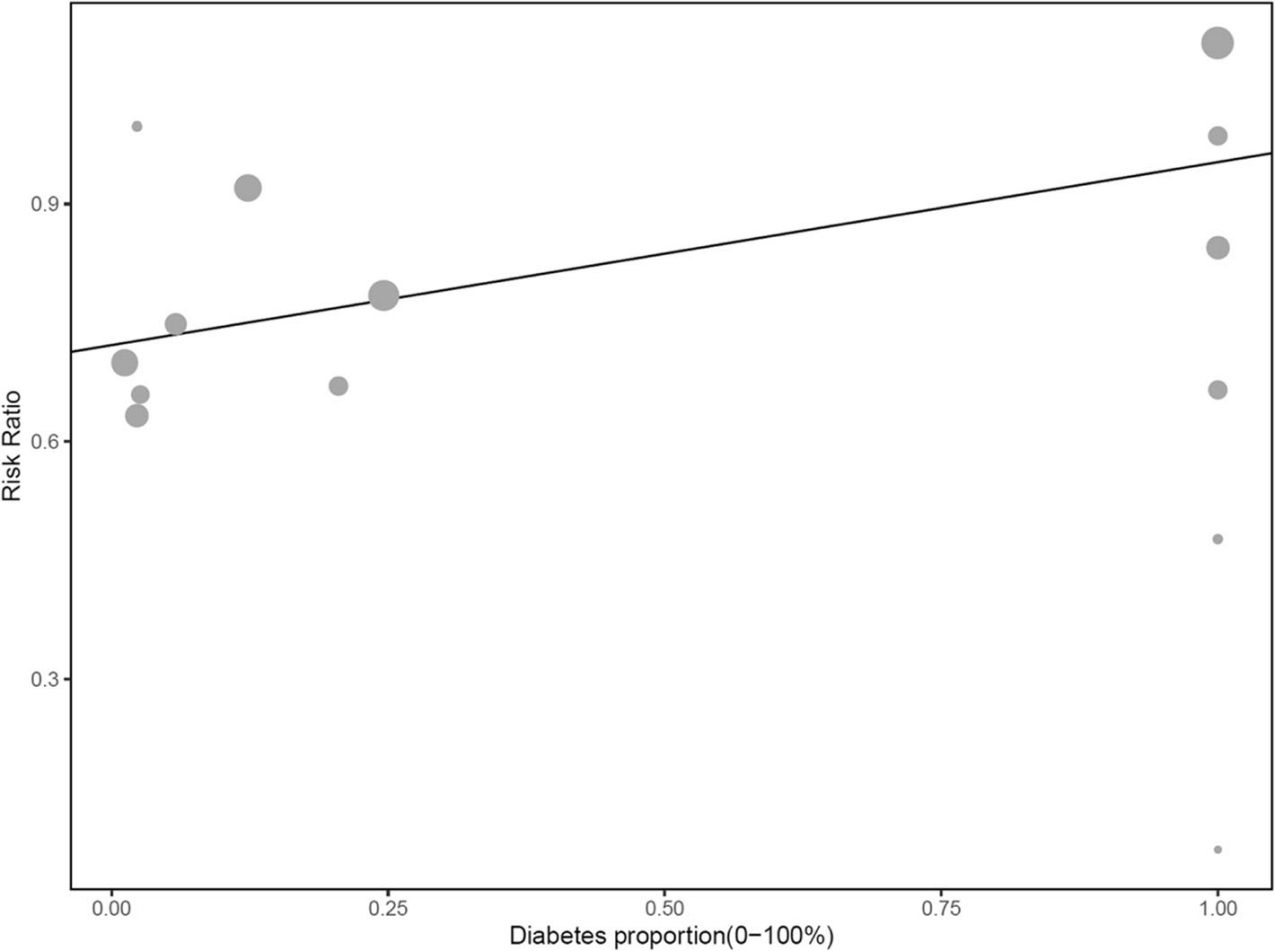
Meta-regression analyses of coronary event risk according to diabetes proportions

The results in stratified baseline LDL-C level for cardiovascular outcomes concurred with the overall estimated results (Fig. 3). Compared with the cardiovascular risk reduction of 19% in LDL-C level of 130 mg/dL or lesser (RR 0.81, 95% CI 0.69–0.97) preferring more intensive lipid-lowering, we found greater cardiovascular risk reduction in higher baseline LDL-C level: 28% in LDL-C level of 160 mg/dL or greater (RR 0.72, 95% CI 0.62–0.84), and 29% in LDL-C level of 130–160 mg/dL (RR 0.71, 95% CI 0.61–0.83). In meta-regressions, cardiovascular outcomes’ RR of the intensive lipid-lowering vs. standard one did not vary by increasing the proportions of women or increasing ages. Figure 4 shows that intensive lipid-lowering for coronary heart disease protection was more pronounced in the non-diabetic populations.

### All-cause mortality

Overall, 2721 of the 46,608 participants (5.84%) receiving more intensive LDL-C-lowering treatment vs. 2922 of the 46,475 participants (6.29%) receiving standard lipid treatment died during follow-up. The risk reduction in all-cause mortality associated with more intensive vs. standard lipid-lowering therapy across all trials was 0.90 (95% CI, 0.83–0.97) (Figure S2) but varied by baseline LDL-C level in the trial. A greater risk reduction was noted with higher LDL-C level (Figure S3). Subgroup-analyses of risk for all-cause mortality of participants in the intensive lipid-lowering group by different definitions showed consistent results with those for cardiovascular outcomes (Figure S4). In meta-regressions, total mortality rates’ RRs of the intensive lipid-lowering vs. standard one did not vary by increasing the proportions of women or increasing ages or diabetes.

### Risk of bias

Potential sources of bias are shown in Table S3 with full qualitative assessments of all RCTs performed and risk of bias judged as low, some concerns, or high risk. Publication bias ranging from none to moderate was suggested using visual inspection of the funnel plots (Figure S5) or the Egger’s test. The evidence of publication bias of cardiovascular outcomes on the Egger’s linear regression test was significant. Sensitivity analyses in Figures S6-1 and S6–2 showed robust results regardless of cardiovascular outcomes or all-cause mortality rate analyses after excluding studies with high risk of bias.

## Discussion

In this study, intensive lipid-lowering caused greater LDL-C reduction than standard lipid-lowering. A greater reduction in the risk of coronary heart disease and all-cause mortality was noted in intensive lipid-lowering among primary prevention settings. The absolute risk reduction (ARR) of intensive lipid lowering in coronary heart disease among studies was approximately between 0.0 and 7.3%. The huge variation in ARR may be attributed to the different coronary risk categories of the selected populations and distinct strategies of intensive lipid-lowering [10, 34]. The pooled number needed to treat (NNT) with intensive lipid-lowering among the included studies was approximately 91 to prevent one coronary heart disease event [35]. In addition, a larger reduction of coronary heart disease risks and all-cause mortality was also found in intensive lipid-lowering for those with higher baseline LDL-C level, particularly for those with LDL-C level of 160 mg/dL or higher. The results indicate that LDL-C level may account for only a part of the total CHD risk. Other residual cardiovascular risk factors also contribute to the progression of CHD [36]. Ceiling effect becomes an issue if treatments only target on LDL-C level control [14, 33, 36]. In our meta-regression results, the effect of intensive lipid-lowering on coronary heart disease and all-cause mortality risk reduction was not different across different sexes or ages. We found that diabetes status was a marginally significant effect modifier between intensive lipid-lowering and coronary heart disease risk reduction. Lipid-lowering effect among non-diabetic populations may be more significant than that among diabetic populations. Our study results may offer a rational explanation for the observations shown in epidemiological studies, to the effect that inflammatory burden, residual lipid-related CVD risk from triglyceride, or apo-lipoproteins, such as apo-lipoprotein CIII, are more pronounced in diabetes population [37]. Based on our subgroup analyses and meta-regression, we found that the source of heterogeneity among the studies was largely attributable to the differing definitions of intensive lipid-lowering treatment (e.g., populations in the studies were exposed to differing LDL-C targets, or there were differing potencies of lipid-lowering agents, including varying intensities of use for statins, fibrate, or combined agents), different baseline LDL-C levels, and diabetes status. Moreover, evidence demonstrated that the heterogeneity in the magnitude of risk reduction of total mortality and coronary heart disease was due to the different baseline cardiovascular risk profiles, including sex, age, smoking rate, smoking, lipid level, and family history of premature cardiovascular mortality [10, 37].

A small study effect by Beishuizen et al. (RR = 0.09, *N* = 182, 20] was noted in publication bias evaluation. Two probable reasons may explain the source of the bias. One is that the coronary events in the study were probably not prespecified study results, raising potential risk of bias, which may cause selective reporting of the most favorable outcomes. The other reason was that the study initially used 0.4 mg cerivastatin but shifted to 20 mg simvastatin in 2001. If we removed Beishuizen et al. from our analyses, evidence of publication bias on the Egger’s linear regression test would be no longer significant whether in coronary heart disease or all-cause mortality analyses.

Treating the LDL-C level to target level or even lower is a major health issue because of its initiating role on atheroma formation. Intensive lipid-lowering strategy has remained an effective method to reduce LDL-C level, but some evidence revealed large interindividual variation [38]. Many epidemiological studies have established larger coronary heart disease risk reduction in intensive lipid-lowering, which was consistent with our study results. However, few of them clarified the effect modifications of sex, age, and diabetes status between intensive lipid treatment and coronary heart disease prevention on elucidating the discrepant efficacy of intensive lipid-lowering in populations. Previous literature about the efficacy of intensive lipid-lowering treatment on all-cause mortality risk reduction remained controversial. Cholesterol Treatment Trialists’ Collaboration found a similar one-tenth risk reduction in all-cause mortality in a 27-trial meta-analysis [10]. However, Ray et al. reported that statin therapy, compared with placebo use, does not have evident benefits on all-cause mortality in a primary prevention cohort [37].

The results of our study show primary care providers that intensive lipid-lowering therapy may be beneficial and necessary in potential medium to high cardiovascular risk populations, such as diabetic and high baseline LDL-C populations, in preventing the first incidence of coronary heart disease and all-cause death. For diabetes groups, in addition to intensive lipid-lowering, sufficient sugar control, other lipid-related risk management, and inflammatory modulation for coronary heart disease risk reduction must not be overlooked [39, 40]. For the non-diabetes group, despite being a relatively lower risk group than the diabetes group, intensive lipid-lowering treatment remains a better choice than standard lipid treatment for preventing incidence of coronary heart disease and all-cause mortality. Although we did not analyze the potential adverse effects of intensive lipid-lowering, some epidemiologic evidence show that intensive lipid-lowering, mainly involving statin use, may cause potential adverse effects such as increasing the risks of new-onset diabetes, myopathy, and liver function impairment. Primary care providers must monitor the development of potential adverse effects while administering intensive lipid-lowering drugs for cardiovascular risk reduction [34, 41].

To our knowledge, our study is the first systematic review to compare the degree of coronary heart disease and all-cause mortality risk reduction among different intensive lipid-lowering strategies specified in primary prevention settings. Our study contributed evidence to intensive lipid-lowering on coronary heart disease and all-cause mortality risk reduction in primary prevention settings. Some limitations were noted in our study. First, our study only offered study-level data and only explored the heterogeneity between studies. Assessing the population cardiovascular risk via study-level data is our limitation; thus, we could not clearly specify the priority group for intensive lipid-lowering in primary prevention settings. Second, new potent lipid-lowering agents were not included because they did not present with solid cardiovascular outcomes nor specified primary preventive outcomes. Third, no quantitative assessment of adverse effect of intensive lipid-lowering was performed in our study.

## Conclusions

In summary, our study indicates that more intensive LDL-C lowering was associated with a greater reduction in risk of CVD and all-cause mortality in trials of patients with higher baseline LDL-C levels. Intensive lipid-lowering among patients without diabetes remains an important strategy for cardiovascular risk reduction. Further studies are urgently needed to clarify the benefits of intensive lipid-lowering on diabetes populations.

## Supplementary information

**Additional file 1:Table S1.** Search strategy. **Table S2.** Revised Cochrane risk-of-bias tool (RoB 2.0) for quality assessment of included RCTs. **Figure S1.** Forest plot of pooled RR for coronary events of participants in the intensive lipid-lowering group by different definitions. **Figure S2.** Forest plot of pooled RR for all-cause mortality of participants in the intensive lipid-lowering group (overall meta-analysis). **Figure S3.** Forest plot of subgroup-analyses of all-cause mortality of participants in the intensive lipid lowering group by different baseline LDL-C levels. **Figure S4.** Forest plot of pooled RR for all-cause mortality of participants in the intensive lipid-lowering group by different definitions. **Figure S5.** Publication bias by funnel plot of coronary events. **Figure S6-1.** Sensitivity analyses of coronary event of participants in the intensive lipid-lowering group**. Figure S6-2.** Sensitivity analyses of all-cause mortality of participants in the intensive lipid-lowering group.

## Data Availability

All data and materials analyzed in the study are presented in the manuscript or supplementary files.

## Abbreviations

RR: Risk ratio
LDL-C: Low-density lipoprotein cholesterol
CVD: Cardiovascular diseases
PRISMA: Preferred reporting items for systematic reviews and meta-analyses
RCT: Randomized controlled trial
CI: Confidence intervals
RoB: Risk of bias
