# Supplementary material for: Efficacy of more intensive lipid-lowering therapy on cardiovascular diseases: a systematic review and meta-analysis
Source: BMC Cardiovasc Disord. 2020 Jul 13;20:334. doi: 10.1186/s12872-020-01567-1 (PMC7359015; doi:10.1186/s12872-020-01567-1)
Supplement: Supplementary file 1 — Additional file 1:Table S1. Search strategy. Table S2. Revised Cochrane risk-of-bias tool (RoB 2.0) for quality assessment of included RCTs. Figure S1. Forest plot of pooled RR for coronary events of participants in the intensive lipid-lowering group by different definitions. Figure S2. Forest plot of pooled RR for all-cause mortality of participants in the intensive lipid-lowering group (overall meta-analysis). Figure S3. Forest plot of subgroup-analyses of all-cause mortality of participants in the intensive lipid lowering group by different baseline LDL-C levels. Figure S4. Forest plot of pooled RR for all-cause mortality of participants in the intensive lipid-lowering group by different definitions. Figure S5. Publication bias by funnel plot of coronary events. Figure S6-1. Sensitivity analyses of coronary event of participants in the intensive lipid-lowering group. Figure S6-2. Sensitivity analyses of all-cause mortality of participants in the intensive lipid-lowering group. [file 12872_2020_1567_MOESM1_ESM.docx]

**Supplementary Materials**

**Table S1. Search strategy.**

Search strategy in Pubmed

| Search | Query |
| --- | --- |
| #16 | Search (((((((((((coronary arteriosclerosis) OR coronary atherosclerosis) OR coronary artery diseases) OR coronary artery disease) OR myocardial ischemia) OR ischemic heart disease) OR heart attacks) OR myocardial infarctions) OR cardiovascular disease) OR cardiovascular disease)) AND ((((((((((cholesterol, ldl OR low density lipoprotein cholesterol OR ldl cholesterol OR ldl cholesteryl linoleate)) OR ((Lipid[Title/Abstract] OR LDL[Title/Abstract] OR Cholesterol[Title/Abstract])))) AND ((lower*[tiab]) OR decreas*[tiab]))) AND ((((((randomized controlled trial[Publication Type]) OR Controlled clinical trial[Publication Type]) OR clinical trial[Publication Type]) OR ((random*[Title/Abstract] AND control*[Title/Abstract] AND trial*[Title/Abstract]))) OR (randomized[Title/Abstract] OR randomly[Title/Abstract] OR placebo[Title/Abstract] OR trial[Title/Abstract] OR crossover procedure[Title/Abstract] OR double blind[Title/Abstract] OR single blind[Title/Abstract]))))) NOT (("Animals"[Mesh]) NOT "Humans"[Mesh]))) |
| #15 | Search (((((((((Coronary Arterioscleroses) OR Coronary Atheroscleroses) OR Coronary Artery Diseases) OR coronary artery disease) OR myocardial ischemia) OR Ischemic Heart Disease) OR Heart Attacks) OR Myocardial Infarctions) OR cardiovascular disease) OR Cardiovascular Disease |
| #14 | Search Coronary Arterioscleroses |
| #13 | Search Coronary Artery Diseases |
| #12 | Search coronary artery disease |
| #11 | Search myocardial ischemia |
| #10 | Search Ischemic Heart Disease |
| #9 | Search Heart Attacks |
| #8 | Search Myocardial Infarctions |
| #7 | Search Infarction, Myocardial Infarctions, Myocardial Myocardial Infarctions Cardiovascular Stroke Cardiovascular Strokes Stroke, Cardiovascular Strokes, Cardiovascular Heart Attack Heart Attacks Myocardial Infarct Infarct, Myocardial Infarcts, Myocardial Myocardial Infarcts |
| #6 | Search Cardiovascular Disease |
| #5 | Search cardiovascular disease |
| #4 | Search (((((((((Cholesterol, LDL OR Low Density Lipoprotein Cholesterol OR LDL Cholesterol OR LDL Cholesteryl Linoleate)) OR ((Lipid[Title/Abstract] OR LDL[Title/Abstract] OR Cholesterol[Title/Abstract])))) AND ((lower*[tiab]) OR decreas*[tiab]))) AND ((((((randomized controlled trial[Publication Type]) OR Controlled clinical trial[Publication Type]) OR clinical trial[Publication Type]) OR ((random*[Title/Abstract] AND control*[Title/Abstract] AND trial*[Title/Abstract]))) OR (randomized[Title/Abstract] OR randomly[Title/Abstract] OR placebo[Title/Abstract] OR trial[Title/Abstract] OR crossover procedure[Title/Abstract] OR double blind[Title/Abstract] OR single blind[Title/Abstract]))))) NOT (("Animals"[Mesh]) NOT "Humans"[Mesh])) |
| #3 | #1 OR #2 |
| #2 | Search (Lipid[Title/Abstract] OR LDL[Title/Abstract] OR Cholesterol[Title/Abstract]) |
| #1 | Search (Cholesterol, LDL OR Low Density Lipoprotein Cholesterol OR LDL Cholesterol OR LDL Cholesteryl Linoleate) (Lipid[Title/Abstract] OR LDL[Title/Abstract] OR Cholesterol[Title/Abstract]) |

**Table S2. Revised Cochrane risk-of-bias tool (RoB 2.0) for quality assessment of included RCTs**

| Domain1 | Risk of bias arising from the randomization process | |
| --- | --- | --- |
| Domain2 | Risk of bias due to deviations from the intended interventions | |
| Domain3 | Risk of bias due to missing outcome data | |
| Domain4 | Risk of bias in measurement of the outcome | |
| Domain5 | Risk of bias in selection of the reported result | |
| Overall risk of bias | Low risk of bias | The study is judged to be at low risk of bias for all domains for this result. |
|  | Some concerns | The study is judged to raise some concerns in at least one domain for this result, but not to be at high risk of bias for any domain. |
|  | High risk of bias | The study is judged to be at high risk of bias in at least one domain for this result. Or  The study is judged to have some concerns for multiple domains in a way that substantially lowers confidence in the result. |

|  | Domain 1 | Domain 2 | Domain 3 | Domain 4 | Domain 5 | Overall risk of bias |
| --- | --- | --- | --- | --- | --- | --- |
| LRC-CPPT, 1984 | Low | Low | Low | Low | Low | Low |
| HHS, 1987 | Low | Low | Low | Low | Some Concerns^a^ | Some Concerns |
| ACAPS, 1994 | Low | Low | Low | Low | Low | Low |
| WOSCOPS, 1995 | Low | Low | Low | Low | Low | Low |
| AFCAPS/TexCAPS, 1998 | Low | Low | Low | Low | Low | Low |
| Sasaki et al., 2002 | High^b^ | Low | Low | Low | Low | High |
| ASCOT-LLA, 2003 | Low | Low | Low | Low | Low | Low |
| Beishuizen et al., 2004 | Low | Low | Low | Low | Low | Low |
| CARDS, 2004 | Low | Low | Low | Low | Low | Low |
| FIELD, 2005 | Low | Low | Low | Low | Low | Low |
| MEGA, 2006 | Low | Low | Low | Low | Low | Low |
| ASPEN, 2006 | Low | Low | Some Concerns^c^ | Low | Some Concerns^a^ | Some Concerns |
| JUPITOR, 2008 | Low | Low | Low | Low | Low | Low |
| Heljic et al., 2009 | Low | Low | Low | Some Concerns | Low | Some Concerns |
| SHARP, 2011 | Low | Low | Low | Low | Low | Low |
| HOPE-3, 2016 | Low | Low | Low | Low | Low | Low |
| EMPATHY, 2018 | High^b^ | Low | High^c^ | Low | Low | High |
| Kitas et al., 2019 | Low | Low | Low | Low | Low | Low |

^a^ Pre-specified analysis plan not mentioned, ^b^ Allocation sequence random and concealment not complete, ^c^ Results likely biased by missing outcome data

Figure S1. Forest plot of pooled RR for coronary events of participants in the intensive lipid-lowering group by different definitions

**
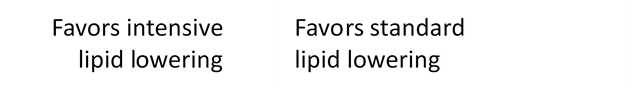
**

**Figure S2.** Forest plot of pooled RR for all-cause mortality of participants in the intensive lipid-lowering group (overall meta-analysis)


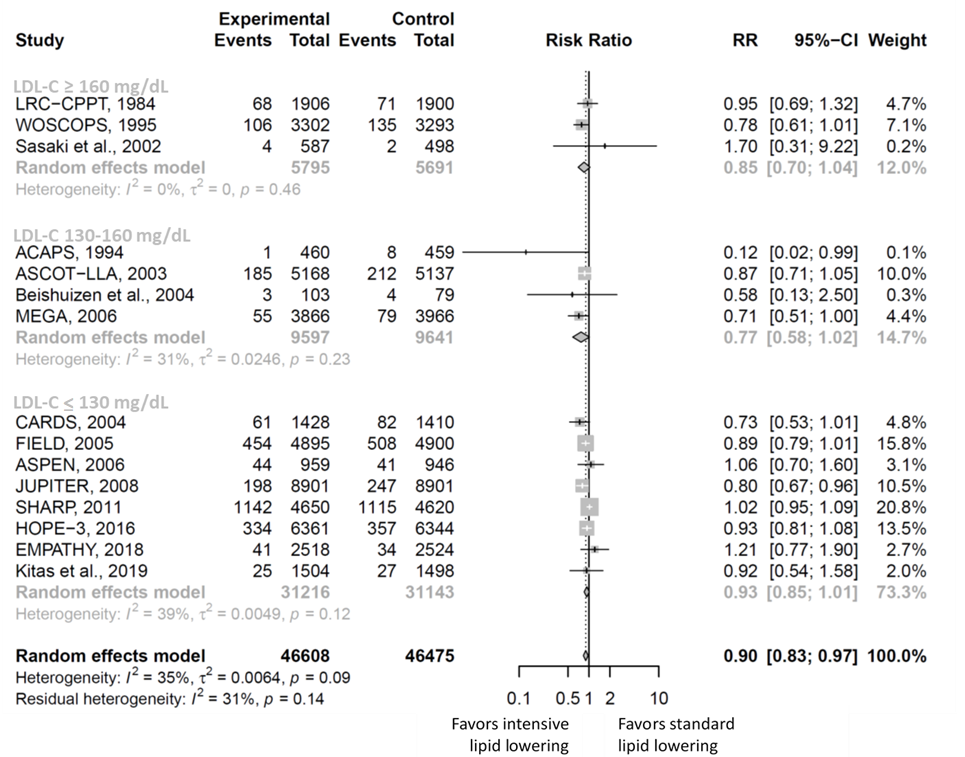


Figure S3. Forest plot of subgroup-analyses of all-cause mortality of participants in the intensive lipid lowering group by different baseline LDL-C levels


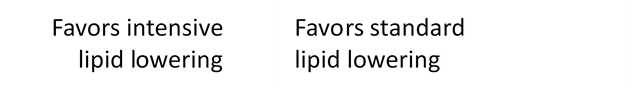

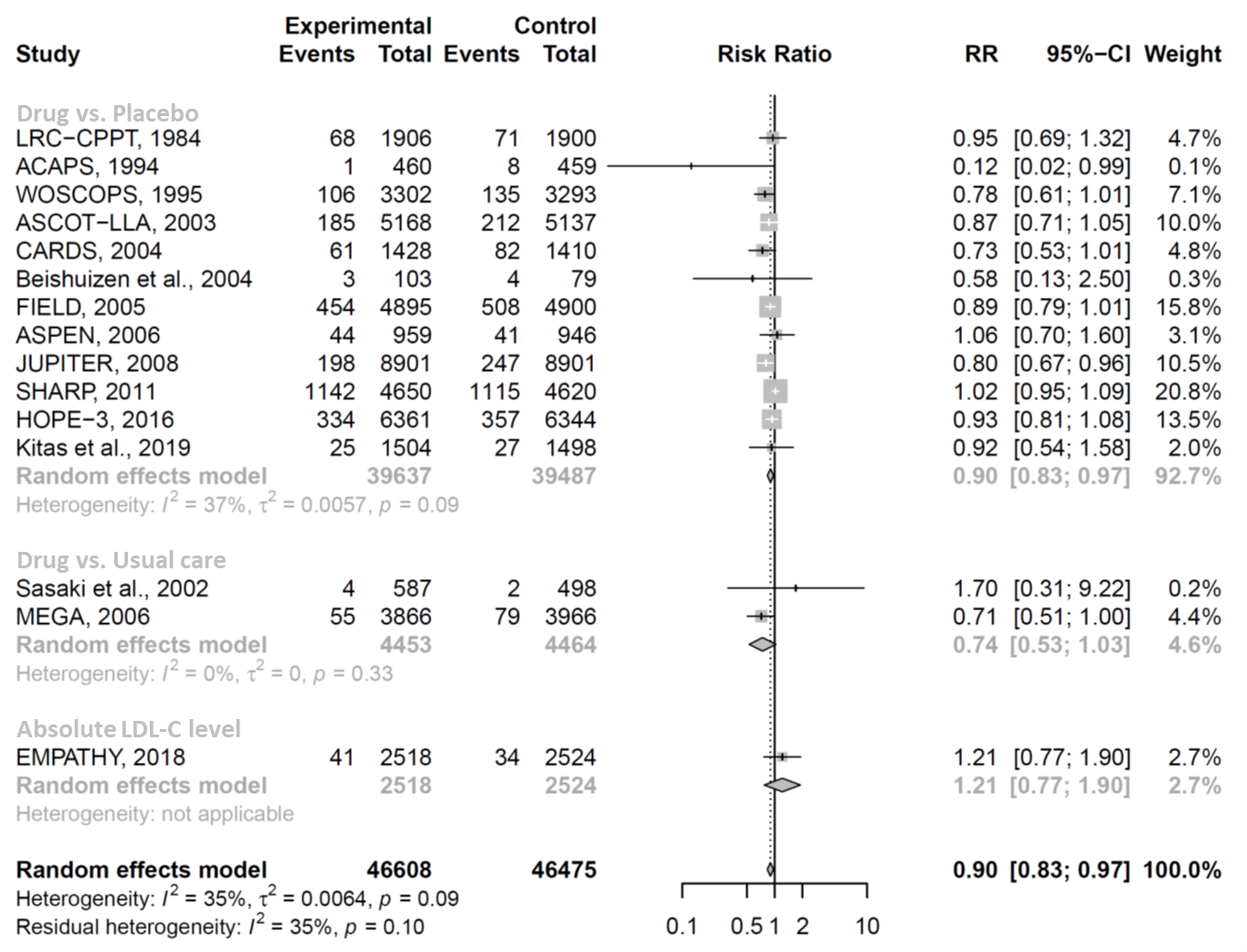


Figure S4. Forest plot of pooled RR for all-cause mortality of participants in the intensive lipid-lowering group by different definitions


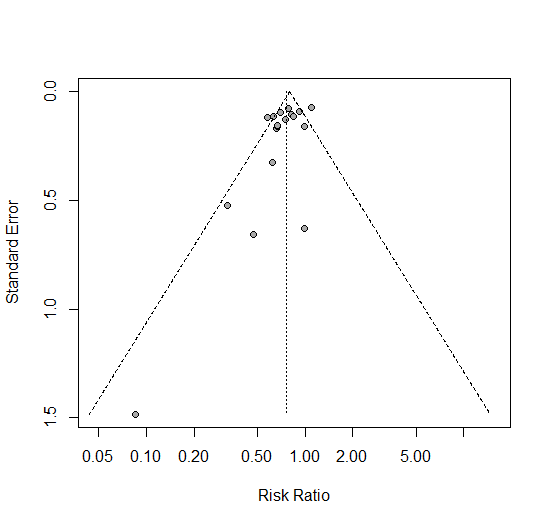


Figure S5. Publication bias by funnel plot of coronary events

Figure S6-2. Sensitivity analyses of all-cause mortality of participants in the intensive lipid-lowering group

Figure S6-1. Sensitivity analyses of coronary event of participants in the intensive lipid-lowering group
